# Customized passive-dynamic ankle–foot orthoses can improve walking economy and speed for many individuals post-stroke

**DOI:** 10.1186/s12984-024-01425-7

**Published:** 2024-07-29

**Authors:** Jacob T. Skigen, Corey A. Koller, Luke Nigro, Darcy S. Reisman, Zahra McKee, Shay R. Pinhey, Adrienne Henderson, Jason M. Wilken, Elisa S. Arch

**Affiliations:** 1 Department of Biomedical Engineering, University of Delaware, Newark, DE USA; 2 Biomechanics and Movement Science Interdisciplinary Program, University of Delaware, Newark, DE USA; 3 Department of Kinesiology and Applied Physiology, University of Delaware, Newark, DE USA; 4 Department of Mechanical Engineering, University of Delaware, Newark, DE USA; 5 Department of Physical Therapy, University of Delaware, Newark, DE USA; 6 Department of Physical Therapy and Rehabilitation Science, University of Iowa, Iowa City, IA USA

**Keywords:** Ankle–foot orthosis, Gait biomechanics, Mechanical cost-of-transport, Walking energy, Poststroke gait

## Abstract

**Background:**

Passive-dynamic ankle–foot orthoses (PD-AFOs) are often prescribed to address plantar flexor weakness during gait, which is commonly observed after stroke. However, limited evidence is available to inform the prescription guidelines of PD-AFO bending stiffness. This study assessed the extent to which PD-AFOs customized to match an individual’s level of plantar flexor weakness influence walking function, as compared to No AFO and their standard of care (SOC) AFO.

**Methods:**

Mechanical cost-of-transport, self-selected walking speed, and key biomechanical variables were measured while individuals greater than six months post-stroke walked with No AFO, with their SOC AFO, and with a stiffness-customized PD-AFO. Outcomes were compared across these conditions using a repeated measures ANOVA or Friedman test (depending on normality) for group-level analysis and simulation modeling analysis for individual-level analysis.

**Results:**

Twenty participants completed study activities. Mechanical cost-of-transport and self-selected walking speed improved with the stiffness-customized PD-AFOs compared to No AFO and SOC AFO. However, this did not result in a consistent improvement in other biomechanical variables toward typical values. In line with the heterogeneous nature of the post-stroke population, the response to the PD-AFO was highly variable.

**Conclusions:**

Stiffness-customized PD-AFOs can improve the mechanical cost-of-transport and self-selected walking speed in many individuals post-stroke, as compared to No AFO and participants’ standard of care AFO. This work provides initial efficacy data for stiffness-customized PD-AFOs in individuals post-stroke and lays the foundation for future studies to enable consistently effective prescription of PD-AFOs for patients post-stroke in clinical practice.

*Trial Registration:* NCT04619043.

**Supplementary Information:**

The online version contains supplementary material available at 10.1186/s12984-024-01425-7.

## Background

Stroke is one of the leading causes of long-term disability, with more than 795,000 individuals in the United States experiencing a stroke each year [1]. Weakened plantar flexor muscles on the paretic limb is a common impairment after stroke [2, 3]. This weakness compromises the individual’s ability to control the lower leg's forward rotation during mid-to-terminal stance [2, 4, 5] and to generate forward propulsion during push-off [2, 5]. This impaired ankle function can result in kinematic deviations during stance including excessive ankle dorsiflexion [6] or persistent knee extension/hyperextension [5, 7]. Impaired ankle function also inhibits forward progression [5] by causing decreased gait speed [2, 5], shorter and asymmetric step lengths [8], and an increased metabolic cost of walking [9–12]. Poor walking economy has been linked to decreases in mobility and participation in daily activities [13, 14], which in turn have been shown to negatively impact both the physical [15–17] and mental [18] well-being of chronic stroke survivors.

Passive-dynamic ankle–foot orthoses (PD-AFOs) are a type of unpowered orthotic device that are gaining popularity for patients with neuromuscular impairments. PD-AFOs can be used to mitigate the negative effects on gait caused by weakened plantar flexors because they have a spring-like bending stiffness [19–22] that provides resistance to help control shank forward rotation during stance-phase dorsiflexion [23]. Additionally, as the PD-AFO deflects during the stance phase, it acts like a torsional spring by storing mechanical energy, which is returned during push-off to aid in forward progression [22]. In this way, PD-AFOs mimic many of the functions of healthy plantar flexor muscles. However, achieving optimal patient outcomes with PD-AFOs likely requires customizing the PD-AFO stiffness to provide personalized support for each individual [19, 24–26].

Despite the recognized need to match AFO characteristics to a patient’s needs [21], there is a lack of objective prescription guidelines to drive this matching for most, if not all, currently prescribed “standard-of-care” (SOC) AFOs. The lack of guidelines often results in an iterative trial-and-error approach to achieve a suitable PD-AFO strut stiffness, and results in substantial inconsistencies in the SOC AFOs that are currently provided. Such inconsistencies lead to varied and often limited patient outcomes for AFO users [24] likely due to a mismatch between orthosis characteristics and a patient’s needs [21].

Prior studies have begun to investigate the effects of PD-AFO stiffness on gait and propose methods for matching PD-AFO stiffness to patients’ needs, but there are still gaps in the knowledge. Pilot studies by our group examined the immediate effects of wearing a PD-AFO with the stiffness customized to make up for each individual’s level of plantar flexor weakness after stroke[19, 27]. The findings suggest customized PD-AFOs can increase the peak paretic plantar flexion moment, but results of other biomechanical and walking performance parameters were inconsistent [27]. Further, the prior studies did not examine more global outcome measures like cost-of-transport (COT) or orthosis satisfaction, and sample sizes were small. Other researchers have demonstrated that customizing PD-AFO stiffness can improve metabolic cost and gait speed compared to walking shod without an AFO and wearing an SOC AFO [24, 25, 28–30]. However, this research used a qualitative decision scheme to select one of five predetermined AFO stiffness values for each participant, based primarily on metabolic cost and gait speed outcomes, rather than utilizing an a priori prescription model. Further, this research did not include individuals with stroke. Multiple studies evaluating the effect of PD-AFO stiffness have been conducted in individuals post-limb salvage [31–33]; however given the many differences between the limb-salvage and post-stroke populations, results cannot be generalized across these populations. Thus, there is still insufficient evidence evaluating efficacy of a standardized, objective method for customizing PD-AFO stiffness to meet the individual needs of persons post-stroke.

The purpose of this study was to evaluate efficacy of stiffness-customized PD-AFOs in reducing total mechanical COT, improving self-selected walking speed (SSWS), improving gait biomechanics, and improving orthosis satisfaction compared to walking shod with no AFO and walking with their SOC AFO for individuals post-stroke. We hypothesized that walking with the PD-AFO would significantly decrease total mechanical COT, increase gait speed, improve gait biomechanics (towards typical), compared to walking with no AFO or their SOC AFO; and increase orthosis satisfaction compared to their SOC AFO. The findings of this study could provide evidence to inform the selection of PD-AFO stiffness and an important step toward establishing a standardized, objective prescription guideline for customizing PD-AFO stiffness to improve outcomes for individuals post-stroke.

## Methods

### Participant recruitment

Based on power calculations performed in G*Power software [34] using available pilot data, we aimed to recruit 32 individuals with chronic (> 6 months post stroke) hemiparetic stroke to take part in this study, which was approved by the University of Delaware Institutional Review Board. Eligible individuals had to have been previously prescribed an AFO by a physician, have at least five degrees of dorsiflexion range of motion, and have paretic plantar flexor weakness. Plantar flexor weakness was defined as paretic peak plantar flexor moment during stance at least 0.15 N m/kg lower than a scaled speed matched typical value. The scaled, speed-matched typical values used in this study were derived from data from able-bodied ambulators walking at a range of speeds. In particular, a regression equation was used to relate ankle moment (scaled by body weight and leg length) and gait velocity across the range of speeds, so a typical, scaled ankle moment value at a comparable speed could be identified for each participant [35]. Exclusion criteria included ataxic gait, neurologic conditions other than stroke, bilateral paresis caused by one or more strokes, an inability to walk outside the home prior to the stroke, an inability to walk for two minutes without assistance from another person during daily living (assistive devices such as a cane were allowed), total joint replacement or other orthopedic problems in the lower limb or spine that limited walking ability, and insufficient cardiovascular health. Participants that could not walk without an orthosis were allowed to participate only if it was an articulating AFO that provided no dorsiflexion resistance and did not have a maximal dorsiflexion limit. Each participant made three visits to the laboratory for this study during which data were collected under three conditions (No AFO, SOC AFO, PD-AFO).

### Experimental protocol

#### Visit 1

During the first visit, all study procedures were explained, and participants signed the informed consent. Participants then completed a 10-m walk test without wearing an orthosis to measure SSWS [36]. If the participant could not walk without an orthosis, the participant completed the test while wearing their SOC AFO if it was a simple hinged AFO and thus did not provide any resistance to motion in dorsiflexion or limit maximal dorsiflexion. If a participant wore a non-hinged AFO and could not walk without the AFO, the participant was withdrawn from the study as we were unable to collect a baseline measure of plantar flexor function during gait, which was needed to customize the stiffness of the PD-AFO.

Next, participants underwent an instrumented gait analysis while shod but without any AFO, walking on an instrumented treadmill set to the speed determined by the 10-m walk test. If participants could not walk without an AFO, they wore their hinged SOC AFO during this gait analysis. The following procedures were used for all gait analyses throughout the study. Participants walked on a split-belt instrumented treadmill (Bertec Corp., Columbus, OH, USA) with a light touch on the handrails, if needed. Kinetic and kinematic data were collected using the treadmill force plates and a 13-camera motion capture system (Qualisys, Goteborg, Sweden). Retroreflective markers were attached to the participant’s pelvis, legs, and feet to track six-degree-of-freedom motion [37]. Kinematic and kinetic data were collected at 240 Hz and 1200 Hz respectively, and filtered at 6 Hz and 25 Hz, respectively, using a 4th order zero-lag Butterworth filter. A minimum of 15 s of walking was collected for each condition. Participants wore a safety harness that provided no body weight support.

Geometric measurements of the participant’s paretic lower limb, including foot length, foot width, location of the metatarsal head axis, shank circumference (widest aspect), and shank length (vertical distance from lateral femoral epicondyle to lateral malleolus) were recorded to enable the fit of the PD-AFO to be customized.

#### PD-AFO stiffness prescription

The data collected in Visit 1 was used to customize the PD-AFO for each participant. Visual 3D software (C-Motion Inc., Germantown, MD, USA) was used to calculate each participant’s net peak paretic plantar flexion moment during stance. The peak plantar flexion moment was scaled by body weight and leg length and averaged across all stance phases collected for each participant. The theory underlying the stiffness prescription model used in this study is that the PD-AFO stiffness is intended to “make up for” the lost paretic ankle plantar flexion moment during stance, with the lost moment defined as the difference between a participant’s unassisted plantarflexion moment and a scaled, speed-matched typical value. This difference was then divided by 12°, which is a typical ankle dorsiflexion excursion during the period of interest [19, 35].

**Fig. 1:**
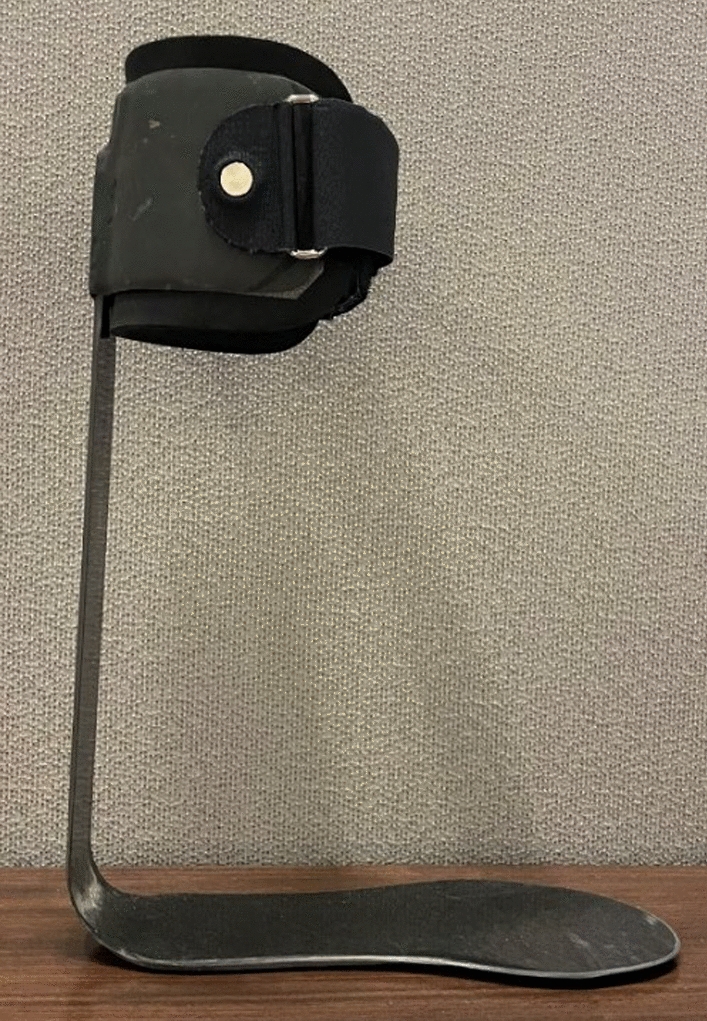
Stiffness-customized carbon fiber PD-AFO

The targeted PD-AFO bending stiffness and limb geometry measurements were provided to our collaborators at the University of Delaware’s Center for Composite Materials, who manufactured a customized PD-AFO for each participant using carbon fiber pre-impregnated with resin (Fig. 1). For each participant, the PD-AFO footplate and cuff were sized based on limb measurements, and PD-AFO strut design was customized via analytic modeling that determined the number of carbon fiber plys and ply orientation to achieve the targeted bending stiffness [38].

#### Visit 2

Once the customized PD-AFO was manufactured, the participant returned to the lab to be fit with the PD-AFO. During this visit, a licensed orthotist assessed, with input from the participant, the PD-AFO footplate and cuff to ensure it fit their leg, was properly aligned when worn inside their shoes, proposed any necessary modifications to the fit of the footplate and cuff, and determined the proper padding and strapping. The orthotist then took the PD-AFO to make any needed modifications. Notably, the orthotist only modified the fit of the footplate and cuff and did not make any modifications that would alter the stiffness of the PD-AFO.

Additionally at this visit, participants underwent an instrumented gait analysis while wearing their SOC AFO while walking at the same speed from Visit 1 (excluding participants who walked with their SOC AFO during Visit 1). After the gait analysis, participants completed another 10-m walk test to record their SSWS while walking with their SOC AFO. Lastly, participants completed the Orthotics and Prosthetics User Survey (OPUS) and Quebec User Evaluation of Satisfaction with assistive Technology (QUEST) Version 2.0 surveys to document their satisfaction with their SOC AFO. Questions regarding orthosis costs and experience with the provider were eliminated from both surveys because they were not relevant to this study. The SOC AFO data were collected at this visit to minimize the burden and fatigue on participants at other visits.

#### Visit 3

During their third visit, participants donned their customized PD-AFO and were given as much time as they needed to acclimate to the PD-AFO by walking around the lab space. Once participants stated they were comfortable walking with the PD-AFO [39], the participants underwent an instrumented gait analysis while wearing the PD-AFO while walking at the same speed as the last two visits. After the gait analysis, participants completed another 10-m walk test to record their SSWS while walking with their PD-AFO. Finally, participants completed the OPUS and Quest 2.0 surveys, now rating their satisfaction with their PD-AFO.

#### Data and statistical analysis

Kinematic and kinetic data were analyzed using Visual 3D and peak paretic dorsiflexion angle, plantar flexion moment, positive ankle power, and positive hip power all during mid-to-late stance were calculated. These ankle measures were chosen as the PD-AFO stiffness prescription model used should, in theory, improve ankle kinematics and kinetics towards typical. Hip power was evaluated to examine if customized PD-AFO use shifted participants from a hip pull-off strategy, which is a common gait compensation seen in individuals with plantar flexor weakness, back to a more typical ankle push-off strategy [40]. These biomechanical parameters for each stance phase were computed using a standard inverse dynamics approach and averaged across trials within each condition (No AFO, SOC AFO, PD-AFO) for each participant. Kinetic variables were scaled by body mass. Additionally, mechanical COT was calculated per limb for each condition as sum of positive limb work (hip, knee, ankle, and distal foot, all normalized by body mass) summed with the absolute value of negative limb work over the gait cycle, scaled by stride length [41]. The COT of both limbs was then combined to calculate the total COT for each participant in each condition. Unlike COT and the biomechanical variables, SSWS values for each condition were determined entirely from overground walking through the 10-m walk tests. Finally, the scores for the OPUS and Quest 2.0 surveys were computed for each condition by assigning a numerical value to each answer option (1–4 for OPUS, 1–5 for QUEST) and totaling the score for each participant. The possible scores ranged from 9 to 36 for OPUS and from 8 to 40 for QUEST.

Descriptive statistics were used to report participant demographics. Group means for each primary outcome variable were calculated in RStudio (Posit team (2023). RStudio: Integrated Development Environment for R. Posit Software, PBC, Boston, MA) by averaging across participants within each condition (No AFO, SOC AFO, PD-AFO). Assumptions of normality and equal variance were evaluated for each group mean using a Shapiro Wilk-W test and 2-sided F-test, respectively. The assumption of equal variance was upheld for all outcome variables. The majority of the primary outcome measures (mechanical COT, SSWS, peak paretic ankle dorsiflexion angle, peak positive ankle power, and peak positive hip power) were normally distributed and thus analyzed using separate repeated measures one-way ANOVAs. However, the peak plantar flexor moment measurements were not normally distributed and as such were analyzed using a Friedman test. When significant main effects were found, post-hoc pairwise comparisons were evaluated with a Holm-Bonferroni correction to account for multiple comparisons. Since the surveys were only collected for the SOC AFO and PD-AFO conditions, they were analyzed differently from the other primary outcome measures. The OPUS responses were normally distributed and therefore analyzed with a paired t-test. However, the QUEST 2.0 responses were not normally distributed, and as such were analyzed using a Wilcoxon Ranked Sign test. Appropriate effect sizes were calculated for all comparisons: omega squared for ANOVAs, Kendall W for Friedman, Cohen’s d for paired t-tests, and common language effect size (CLES) for Wilcoxon Ranked Sign test.

Given the heterogeneity of the post-stroke population, simulation modeling analysis (SMA) was also used to examine results within each participant across conditions to determine if meaningful results were seen on an individual basis that may have been obscured in the group mean analysis due to averaging across participants. SMA was conducted for all primary outcome measures except SSWS, as only one data point was collected for SSWS for each condition, and achieving best results with SMA requires three to eight data points. Thus, minimum detectable change (MDC) was used to analyze SSWS instead of SMA. Minimum detectable change was classified according to participant’s baseline SSWS using previously established values: an individual with a baseline speed less than 0.40 m/s needed to change by at least 0.10 m/s to be deemed a meaningful change, a baseline between 0.40–0.8 m/s required a change of at least 0.15 m/s, and a baseline greater than 0.8 m/s necessitated a change of 0.18 m/s [42].

## Results

### Participants

In total, 112 participants were screened by phone for eligibility and interest in participating in this study. Out of those screened, 27 were not interested in participating in the study and 52 did not qualify for participation based on the inclusion/exclusion criteria (Supplementary Table 1). Thus, 33 participants came into the lab for Visit 1 and were enrolled in this study. A total of 12 participants dropped out or withdrew from this study before completion of Visit 3 and therefore never walked with a customized PD-AFO. Of this 12, four were unable to walk without a rigid brace during their evaluation visit, three were prescribed devices with stiffnesses greater than we had the capability to manufacture, two did not have sufficient plantar flexor weakness to be included, one did not like the type of brace, one could not be comfortably fit, and one did not respond to follow-up contact attempts. Further, Visit 3 data from one participant could not be used due to a technical issue during collection. Thus, data from 20 participants are reported in this study. Relevant demographic and clinical information of these participants are presented in Table 1. Individual participants’ level of plantar flexor weakness, the prescribed stiffness of their customized PD-AFO, and (where possible) the stiffness of their SOC AFO are presented in Table 2.

**Table 1:** Participant demographics

Paretic side (L/R)	8/12
Sex (M/F)	14/6
Age (years)	63.5 ± 10.1
Height (m)	1.74 ± 1.1
Mass (kg)	88.2 ± 17.7
Stroke onset (months)	52.5 ± 25.8

**Table 2:** Individual participants’ SOC AFO type, PF weakness, prescribed customized PD-AFO stiffness, and SOC AFO stiffness

	Participant	SOC AFO	PF weakness	Prescribed PD-AFO stiffness	SOC AFO stiffness
			(% of typical)	(N*m/deg)	(N*m/deg)
Subgroup 1	1	Solid	8.1	0.9	–
	2	PD-AFO	39.5	3.5	^a^
	3	Solid	26.0	1.9	–
Subgroup 2	4	PD-AFO	25.8	2.0	^a^
	5	PD-AFO	15.8	1.1	1.30
	6	PD-AFO	15.3	1.8	0.73
	7	PD-AFO	35.0	3.3	2.12
	8	Hinged	10.6	0.7	0
Subgroup 3	9	Hinged	35.9	3.1	0
	10	Hinged	14.7	1.2	0
	11	Hinged	23.7	2.3	0
	12	Hinged	24.3	2.9	0
	13	–	9.5	0.8	–
	14	–	10.9	1.6	–
	15	PD-AFO	60.4	3.6	0.22
	16	PD-AFO	9.3	0.9	0.86
	17	PD-AFO	19.7	1.8	0.45
	18	Solid	54.5	3.5	–
	19	Hinged	9.4	2.4	0
	20	Hinged	55.9	3.4	0

^a^Represents instances where the participant’s SOC AFO was a passive-dynamic device that we could not measure the stiffness of due to their design/shape

### Group mean results

**Fig. 2:**
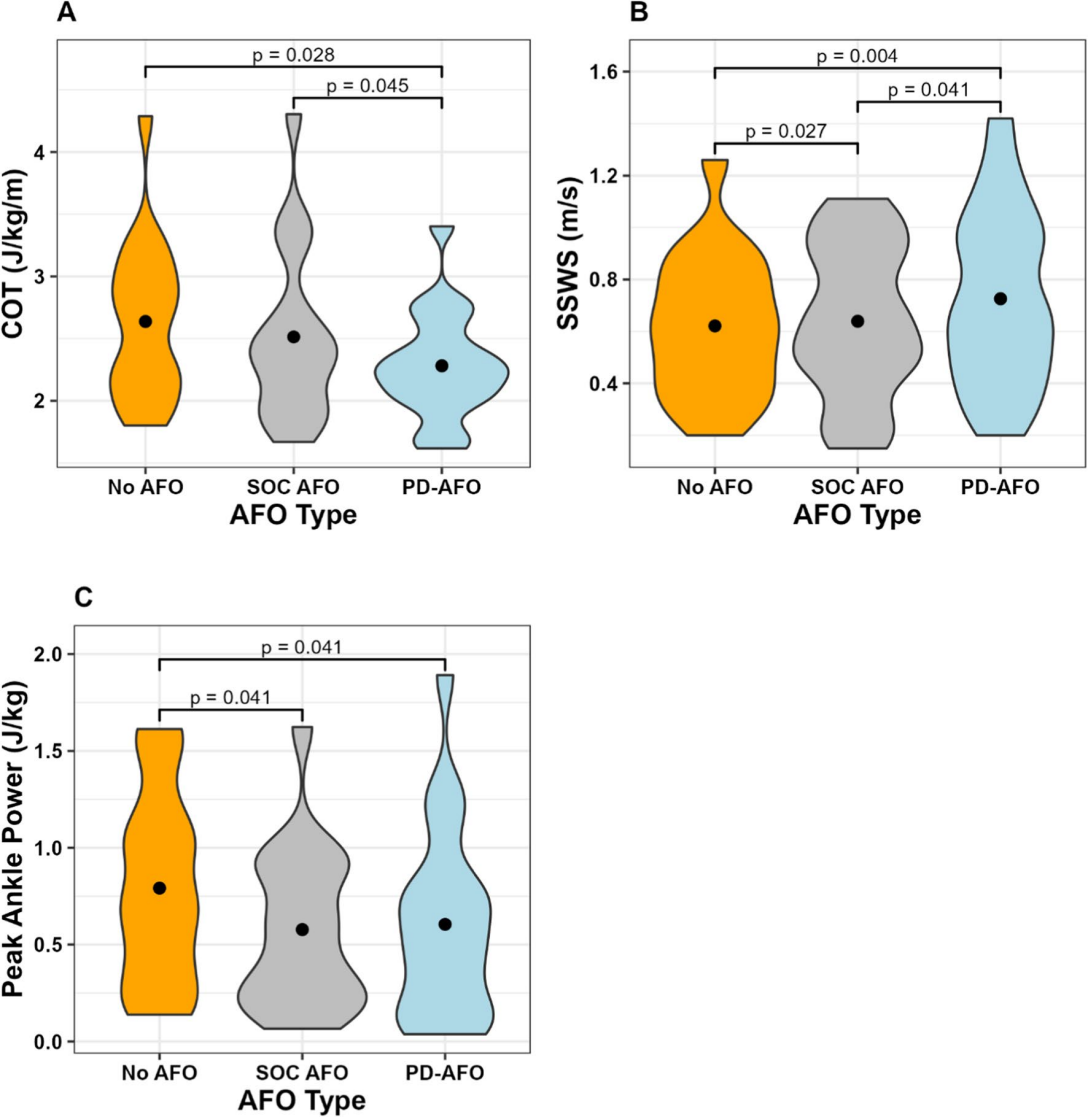
Density plots of significant outcomes. **A** Mechanical cost-of-transportation (J/kg/m). **B** Self-selected walking speed (m/s). **C** Peak positive ankle power (J/kg)

**Fig. 3:**
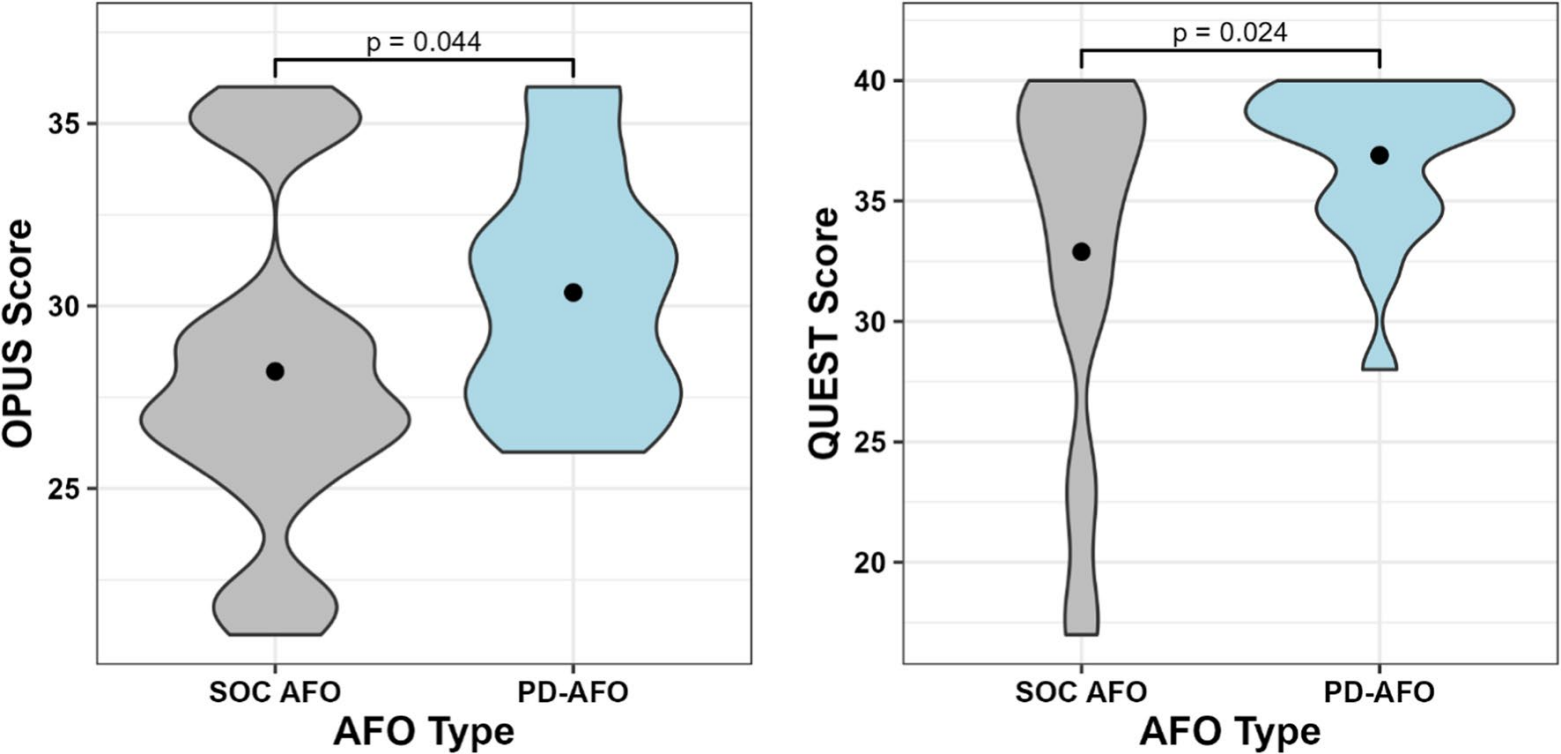
Density plots of participant scores in the OPUS (left) and QUEST (right) surveys

One participant could not walk without their hinged SOC AFO and therefore no data were collected for the No AFO condition. Two participants did not bring their SOC AFO into the lab, so no SOC AFO data were collected for these two participants. Results showed a significant main effect of orthosis condition on COT, SSWS, and peak positive ankle power (Table 3). Figure 2 shows there was a significant reduction in COT between No AFO and PD-AFO (p = 0.028, d = 0.83) and between SOC AFO and PD-AFO (p = 0.045, d = 0.3). The difference between COT in the No AFO and PD-AFO conditions ranged from + 0.15 to − 2.52 J/kg/m with a mean ± SD of − 0.44 ± 0.63. The difference between COT in the SOC AFO and PD-AFO conditions ranged from + 0.14 to − 0.98 J/kg/m with a mean ± SD of − 0.22 ± 0.37. There was also a significant increase in SSWS between No AFO and SOC AFO (p = 0.027, d = 0.32), No AFO and PD-AFO (p = 0.004, d = 0.39), and SOC AFO and PD-AFO (p = 0.041, d = 0.15). Finally, there was a significant decrease in peak positive ankle power between No AFO and SOC AFO (p = 0.041, d = 0.25) and No AFO and PD-AFO (p = 0.041, d = 0.33). QUEST scores for the PD-AFO ranged from 28 to 40 with an average score of 36.9; and OPUS scores ranged from 26 to 36 with an average score of 30.4 (Fig. 3). The average participant score for both the OPUS and QUEST 2.0 surveys significantly improved for the PD-AFO compared to their SOC AFO (Table 3).

**Table 3:** Group means and standard deviations of primary outcome variables for each condition

	No AFO	SOC AFO	PD-AFO	p value	Effect size^a^
COT (J/kg/m)	2.64 ± 0.63	2.51 ± 0.69	2.28 ± 0.42	0.0062^▲■^	0.11
SSWS (m/s)	0.62 ± 0.27	0.64 ± 0.29	0.73 ± 0.34	0.0003^♦▲■^	0.03
Peak DF angle (deg)	16.97 ± 5.95	16.14 ± 4.81	17.37 ± 5.55	0.317	0.0013
Peak PF moment (N*m)	0.93 ± 0.22	0.90 ± 0.25	0.92 ± 0.25	0.662	0.0242
Peak ankle power (J/kg)	0.79 ± 0.48	0.58 ± 0.41	0.60 ± 0.49	0.0117^♦▲^	0.02
Peak hip power (J/kg)	0.38 ± 0.14	0.34 ± 0.15	0.40 ± 0.20	0.275	0.0041
OPUS Score	–	28.25 ± 4.35	30.37 ± 3.15	0.0443^■^	0.55
QUEST 2.0 Score	–	32.95 ± 7.27	36.89 ± 3.21	0.0244^■^	73.68

^a^Type of effect size varies based on statistical test run (see methods)

^♦^Denotes a significant difference between No AFO and SOC AFO condition

^▲^Denotes a significant difference between No AFO and PD-AFO condition

^■^Denotes a significant difference between SOC AFO and PD-AFO condition

### Individual participant results

Individual-level analysis indicated there were significant differences between the three conditions for many participants (Tables 4, 5). SMA results could not be calculated for Participant 3’s SOC condition due to insufficient data with their SOC AFO. While the average COT significantly decreased when walking with the PD-AFO, this result was not observed for all individuals. COT was reduced with the PD-AFO compared to No AFO for all but three individuals; however, the changes were only significant for ten individuals. Similarly, while 12 participants decreased their COT with the PD-AFO compared to their SOC AFO, only six of those were significant. Because not all participants have data for all three conditions it is difficult to compare these groups to one another; however, there were three participants who significantly improved COT with the PD-AFO compared to both No AFO and SOC AFO. For SSWS, nine participants showed a meaningful increase while walking with the PD-AFO compared to walking with No AFO. Three of these participants also meaningfully increased their SSWS compared to walking with their SOC AFO. However, upon visual inspection of the data in Tables 4 and 5, no discernable pattern emerged between these reductions in COT or SSWS and the biomechanical outcome variables analyzed where a significant change in one variable was always matched with a significant change in another. Furthermore, results from the biomechanical variables were inconsistent both within and between participants. While scattered changes were seen, significant and beneficial biomechanical changes were limited. No participant had significant, beneficial changes across all variables with PD-AFO use, and no single variable showed significant improvement with PD-AFO use across even a majority of the participants.

**Table 4:** Individual PD-AFO vs. No AFO key outcome measures

	Participant	Total COT (J/kg/m)		SSWS (m/s)			Peak DF angle (deg)		Peak PF moment (N*m)		Peak ankle power (J/kg)		Peak hip power (J/kg)	
		Δ	R	Base	Δ	MDC	Δ	R	Δ	R	Δ	R	Δ	R
Subgroup 1	1	0.15*	0.82*	0.88	0.09	0.18	− 0.9	− 0.6	0.16*	0.89*	− 0.53*	− 0.85*	− 0.03	− 0.3
	2	0.22	0.66	0.41	− 0.1	0.15	**− 2.6**	**− 0.9**	− 0.05	− 0.32	− 0.08	− 0.84	− 0.02	0.17
	3	0.12	0.18	0.2	0	0.15	− 1.9	− 0.4	0.1	0.34	− 0.07	− 0.61	0.06*	0.7*
Subgroup 2	4	**− 2.52**	**− 0.94**	**0.52**	**0.32**	**0.15**	− 1.2	− 0.6	**− 0.25**	**− 0.94**	0.02	− 0.33	0	− 0.6
	5	**− 1.09**	**− 0.98**	**0.3**	**0.15**	**0.1**	**− 7.6**	**− 1**	0.06	0.66	− 0.14	− 0.84	0.04	0.6
	6	**− 0.46**	**− 0.94**	**0.65**	**0.29**	**0.15**	3.9*	0.9*	0.12*	0.77*	− 0.57*	− 0.96*	− 0.01	− 0.35
	7	**− 0.61**	**− 0.93**	**0.9**	**0.23**	**0.18**	− 0.8	− 0.5	**− 0.38**	**− 0.77**	0.22	0.52	− 0.03	− 0.47
	8	− 0.14	− 0.08	0.56	0.08	0.15	1.7	0.8	0.07*	0.64*	− 0.2*	− 0.75*	0.19	0.62
Subgroup 3	9	− 0.18	− 0.45	0.42	0.06	0.15	**− 1.4**	**− 0.5**	0	0.24	− 0.19*	− 0.9*	0.1*	0.8*
	10	**− 0.28**	**− 0.88**	0.74	− 0.08	0.15	6.6*	1*	**− 0.19**	**− 0.96**	0.08	0.42	**− 0.22**	**− 0.96**
	11	**− 0.25**	**− 0.83**	**0.63**	**0.43**	**0.15**	1.4	0.5	**− 0.14**	**− 0.79**	0.06	− 0.24	0	− 0.1
	12	− 0.12	− 0.34	**0.96**	**0.28**	**0.18**	**− 2.9**	**− 0.9**	− 0.07	− 0.23	− 0.29	− 0.64	− 0.1	− 0.63
	13	− 0.11	− 0.28	0.8	− 0.04	0.15	4.8*	0.9*	0.12	0.67	− 0.26*	− 0.81*	**− 0.08**	**− 0.47**
	14	**− 0.65**	**− 0.91**	1.26	0.16	0.18	2.7	0.5	− 0.13	− 0.28	0.3	0.09	0.03	0.09
	15	**− 1.03**	**− 0.95**	**0.29**	**0.22**	**0.1**	− 0.6	− 0.3	− 0.18	− 0.74	− 0.1	− 0.74	0	− 0.01
	16	**− 0.36**	**− 0.94**	**0.78**	**0.18**	**0.15**	− 0.4	− 0.4	0.18*	0.96*	− 0.63*	− 0.96*	0.01	0.05
	17	**− 0.27**	**− 0.89**	0.65	− 0.06	0.15	− 0.3	− 0.1	0.16*	0.91*	− 0.12	− 0.78	0.13	0.51
	18	–	–	0.36	0.03	0.1	-3.3	-0.6	0.1	0.54	-0.1*	-0.77*	-0.02	-0.16
	19	− 0.26	− 0.63	**0.5**	**0.18**	**0.15**	7.4*	1*	0.06	0.48	-0.4*	-0.97*	-0.06	-0.24
	20	–	–	–	–	–	–	–	–	–	–	–	–	–

Bold text indicates a significant beneficial change (decrease in COT, increase in SSWS, towards typical for biomechanical variables) determined by SMA

*Indicates non-beneficial significant change. Due to a technical issue, mechanical COT could not be calculated for Participant 18’s No AFO condition

**Table 5:** Individual PD-AFO vs. SOC AFO key outcome measures

	Participant	SOC AFO	Total COT (J/kg/m)		SSWS (m/s)			Peak DF angle (deg)		Peak PF moment (N*m)		Peak ankle power (J/kg)		Peak hip power (J/kg)	
			Δ	R	Base	Δ	MDC	Δ	R	Δ	R	Δ	R	Δ	R
Subgroup 1	1	Solid	0.14*	0.87*	0.92	0.05	0.18	1	0.61	**− 0.11**	**0.82**	− 0.54	− 0.83	0.03	− 0.27
	2	PD-AFO	**− 0.2**	**− 0.83**	0.23	0.08	0.1	− 2.8*	− 0.97*	**− 0.12**	**0.65**	0.03	0.64	− 0.11	− 0.37
	3	Solid	− 0.16	−	0.15	0.05	0.1	− 1.4	−	− 0.03	−	− 0.04	–	0	–
Subgroup 2	4	PD-AFO	0.1*	0.77*	**0.63**	**0.21**	**0.15**	2.4*	0.93*	0	0.4	**0.15**	**0.83**	− 0.01	− 0.37
	5	PD-AFO	0.02	0.29	0.5	− 0.05	0.15	**− 3.6**	**− 0.94**	− 0.01	0.12	− 0.05	− 0.46	0.1*	0.94*
	6	PD-AFO	0.05	0.44	0.87	0.07	0.18	3*	0.99*	− 0.03	0.1	− 0.13	− 0.71	− 0.05	− 0.09
	7	PD-AFO	0.26	0.72	1.11	0.02	0.18	− 0.7	− 0.49	0.15	− 0.57	0.36	0.76	0.15	0.47
	8	Hinged	0.03	0.25	0.63	0.01	0.15	− 1.1	− 0.51	**− 0.15**	**0.81**	− 0.16	− 0.69	0.07	0.21
Subgroup 3	9	Hinged	− 0.16	− 0.49	0.48	0	0.15	**− 4.3**	**− 0.42**	0.02	0.07	− 0.06	0.75	0.15*	0.94*
	10	Hinged	**− 0.23**	**− 0.97**	0.68	− 0.02	0.15	1.9	0.26	0.1*	− 0.77*	− 0.14	− 0.24	0.02	− 0.25
	11	Hinged	**− 0.11**	**− 0.53**	1.02	0.04	0.18	4.3*	0.98*	0.16*	− 0.78*	0.37	0.59	0.06	0.5
	12	Hinged	− 0.23	− 0.62	**1.03**	**0.21**	**0.18**	1.9	0.78	− 0.11	0.61	− 0.14	− 0.57	− 0.04	− 0.17
	13	–	–	–	–	–	–	–	–	–	–	–	–	–	–
	14	–	–	–	–	–	–	–	–	–	–	–	–	–	–
	15	PD-AFO	**− 0.98**	**− 0.96**	0.51	0	0.15	3.6*	0.93*	0.22	− 0.8	− 0.09*	− 0.86*	0.09	0.63
	16	PD-AFO	− 0.01	− 0.14	0.91	0.05	0.18	5*	0.98*	− 0.03	0.67	− 0.29*	− 0.94*	0.01	− 0.13
	17	PD-AFO	− 0.14	− 0.22	0.74*	− 0.15*	0.15*	0.1	− 0.21	− 0.07	0.46	0.04	0.77	0.18	0.61
	18	Solid	**− 0.89**	**− 0.93**	0.37	0.02	0.1	− 4.3*	− 0.98*	− 0.05	0.34	− 0.13*	− 0.96*	− 0.01	0.07
	19	Hinged	− 0.55	− 0.77	**0.5**	**0.18**	**0.15**	9.6*	0.98*	− 0.13	0.79	− 0.45*	− 0.93*	− 0.1	− 0.54
	20	Hinged	**− 0.9**	**− 0.79**	0.23	0.06	0.1	3.3*	0.72*	− 0.05	0.13	− 0.15	0.52	0.01	0.56

Bold text represents a significant beneficial change (decrease in COT, increase in SSWS, towards typical for biomechanical variables) determined by SMA

*Indicates non-beneficial significant change

**Fig. 4:**
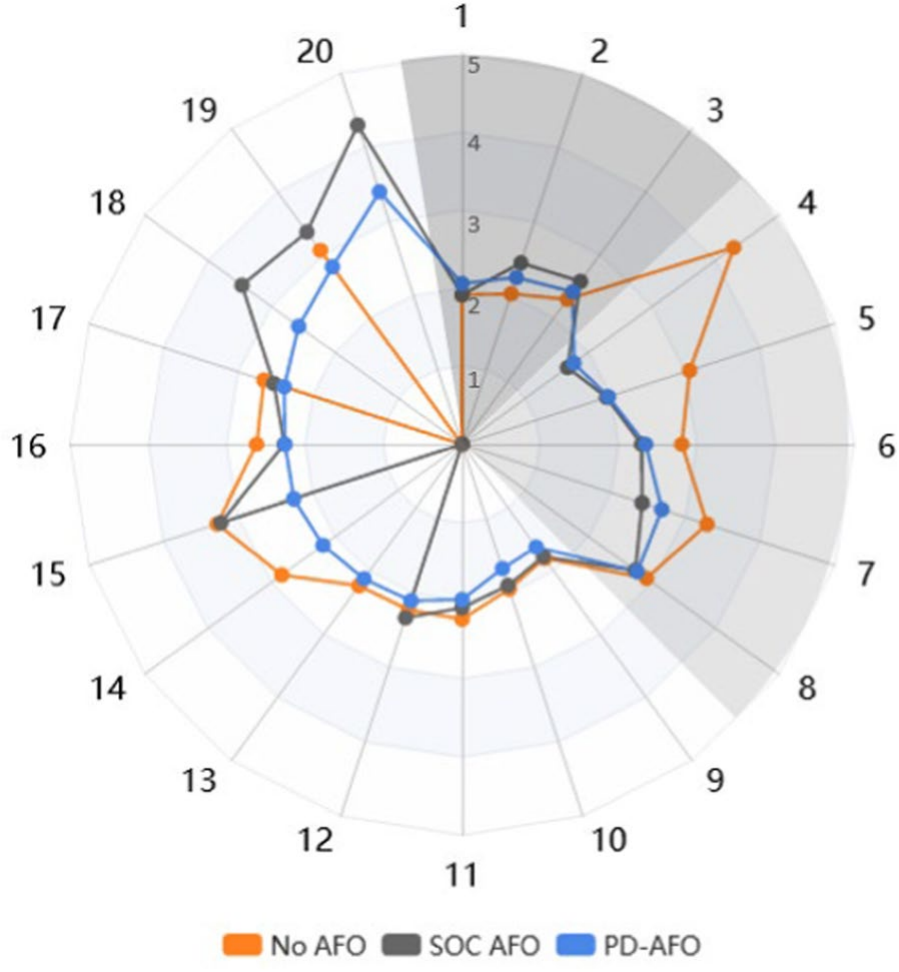
Mechanical COT (J/kg/m) values across all conditions. Subgroup 1 in dark grey, Subgroup 2 in light grey, and Subgroup 3 in white

To further analyze the individual results, participants were categorized according to which condition elicited the lowest mechanical COT (Tables 4, 5). Subgroup 1 (three participants) had the lowest COT while walking with No AFO; Subgroup 2 (five participants) had the lowest COT while walking with their SOC AFO; and Subgroup 3 (twelve participants) had the lowest COT while walking with the PD-AFO. When comparing the average COT values for each participant, Subgroup 1 showed only a small difference in COT across all three conditions, and Subgroup 2 displayed a small difference in COT between the SOC and PD-AFO conditions compared to the No AFO condition. Notably, Subgroup 3 showed a much smaller distribution of average COT values while wearing the PD-AFO compared to the No AFO and SOC AFO conditions (Fig. 4). The PD-AFO did not meaningfully change SSWS for any individuals in Subgroup 1. Likewise, most of Subgroup 2 (notably, the participants who wore PD-AFOs as their SOC AFO) saw significant improvement in COT when walking with the stiffness-customized PD-AFO compared to walking with No AFO. However, when compared to their SOC device, only one participant had a meaningful improvement in SSWS with the PD-AFO. Two participants in Subgroup 3 had a meaningful improvement in SSWS with the PD-AFO compared to their SOC AFO, which is fewer than the number of participants in this group that had a significant improvement in COT with the PD-AFO compared to the SOC AFO.

## Discussion

The purpose of this study was to determine the effect of stiffness-customized PD-AFOs on reducing mechanical COT, increasing SSWS, and improving biomechanical variables, as well as overall orthosis satisfaction, compared to walking without an AFO and to walking with a “standard-of-care” AFO prescribed by clinicians (SOC AFO). On a group level, results showed significant improvements in both COT and SSWS with PD-AFO use, though the biomechanical measures were more variable. However, analysis on an individual level revealed that subgroups of participants responded differently to the PD-AFO.

### Cost of transport

The hypothesis that walking with a stiffness-customized PD-AFO would result in a lower mechanical COT was supported. On a group level, average COT was significantly lower while wearing the stiffness-customized PD-AFO compared to walking with No AFO or a SOC AFO. These findings align with previous research in other populations showing orthosis stiffness can influence walking energetics [25, 26, 28, 43]. Published research in this area typically used metabolic, and not mechanical, cost of transport so it is difficult to directly compare magnitudes of results. Additionally, the density of COT magnitudes was notably more concentrated towards the lower end of the spectrum with the PD-AFO compared to both No AFO and SOC AFO (Fig. 2). This finding emphasizes that many participants experienced lower COT with PD-AFO use. However, the fact that not all participants saw a significant reduction in COT indicates the utility of categorizing the participants based on which condition produced the lowest COT and analyzing them on an individual basis to provide additional context.

There were three individuals in Subgroup 1, which included individuals who had the lowest COT in the No AFO condition. These were the only three individuals who did not reduce their COT with the PD-AFO compared to No AFO Notably, however, when looking at COT magnitudes, all three of these participants had similar COT magnitudes across all three conditions (Fig. 4). Possible explanations for these findings include that these individuals did not really need an orthosis, or they may not have figured out how to effectively use the orthoses.

Subgroup 2 included those who had the lowest COT with their SOC AFO. Four out of five of these individuals were prescribed passive-dynamic devices as their SOC AFO, and most of them significantly reduced their COT when walking with the stiffness-customized PD-AFO compared to No AFO but not compared to their SOC devices. It is possible that these participants had limited capacity for improvement when using the stiffness-customized PD-AFO compared to their SOC AFO because their SOC AFO was already a passive-dynamic device that may have been very similar to the ones provided in this study.

Finally, Subgroup 3, those who walked most efficiently with the stiffness-customized PD-AFO, was the largest subgroup. Unlike Subgroup 2, who all had similar COT values walking with the PD-AFO and their SOC AFO but a higher COT with No AFO, Subgroup 3 did not exhibit a clear trend when comparing the different conditions. While some had similar COT values for all conditions, others had noticeably lower COT with the PD-AFO compared to No AFO or SOC AFO walking; some participants in this subgroup even had their highest COT while walking with their SOC AFO. Subgroup 3 is likely the driving source of the significant COT reductions with the PD-AFO seen at the group level (Fig. 3). Notably, the majority of this subgroup had been prescribed hinged AFOs as their SOC AFO, though there was one participant with a passive-dynamic SOC AFO that significantly lowered their COT while walking with the stiffness-customized PD-AFO. Given the differences between the changes in COT for these subgroups, it is clear that there is no one-size-fits-all approach to prescribing AFOs to lower COT for individuals post-stroke. Future research should delve further into this area to investigate for whom stiffness-customized PD-AFOs are ideal, and if there are patient characteristics that indicate which type of AFO is ideal for a given individual.

### Self-selected walking speed

The hypothesis that walking with a stiffness-customized PD-AFO would result in a faster SSWS was supported. In general, when comparing SSWS across conditions, a similar pattern as was observed in the COT results emerged. On a group level, there was a significant improvement when walking with the PD-AFO compared to both the No AFO and SOC AFO conditions at a level similar to or exceeding that seen in previous research comparing stiffness-optimized orthoses to those prescribed using traditional methods [24, 26]. However, the density plot indicated that there was a wide distribution of SSWS for all three conditions, unlike what was seen with COT (Fig. 2). This finding highlights the variability in SSWS results and a lack of universal improvement for all participants, which was confirmed when the individual results were examined. The fact that on an individual level the majority of participants did not meaningfully change their SSWS is consistent with other studies with varying AFO conditions [24, 28, 44–46] which saw little to no change in walking speed across brace conditions. The lack of a noticeable pattern for how individuals in each subgroup changed their SSWS with the PD-AFO implies that speed would not be a useful measurement for determining which subgroup an individual would fall into. Overall, even though there was significant improvement in SSWS with PD-AFO use on a group level, the individual-level analysis showed SSWS results varied substantially across participants—even more than COT results did, especially for the PD-AFO to SOC AFO comparison. It is possible that SSWS is less sensitive to orthosis condition or changes in walking speed may not appear as readily with immediate use of a new orthosis (i.e. longer-term PD-AFO use may generate greater changes in SSWS). Future research should investigate these possibilities further.

### Biomechanical variables

Our hypothesis that PD-AFO use would result in improved (toward typical) gait biomechanics was not supported. On a group level, the only biomechanical variable that showed a significant difference was peak positive ankle power, which significantly decreased with PD-AFO use compared to walking with No AFO. This decreased ankle power is not a beneficial change, but it is likely a consequence of the device design since PD-AFOs are very stiff in plantar flexion, thereby minimizing plantar flexion motion. While this design feature prevents foot drop, which is another common impairment post stroke, it also makes it difficult for the PD-AFO user to plantar flex and generate push-off power in late stance. The energy storage and return feature of the PD-AFO should provide some positive power about the ankle in late stance, but it was not enough to counteract the limited plantar flexion range of motion for these participants. Notably, a significant decrease in peak positive ankle power was also present when participants walked with their SOC AFO, all of which also arrested ankle plantar flexion past neutral to prevent toe drop.

The underlying theory that peak plantar flexor moment would improve with the stiffness-customized PD-AFOs was not seen for all participants. This finding may be explained in a few ways. First, the theorized prescription model used in this study may not be optimal, at least for some individuals post-stroke. Future work should investigate if different PD-AFO stiffnesses provide more benefits. Alternatively, given the lack of change in peak dorsiflexion angle and hip power across conditions, it is possible that some participants did not fully engage the spring-like bending mechanism of the PD-AFOs. Without full engagement of the PD-AFO, the orthosis could not provide full benefit to the user, which may explain why the theorized mechanism used to prescribe the PD-AFO stiffness was not fully upheld. Future research should investigate if outcomes change if participants engage the PD-AFO more.

The finding of significant changes in SSWS and COT were not reflected in systematic changes in biomechanical measures. Previous studies have found a similar lack of significant change in biomechanical variables on a group level while participants wore a stiffness-customized AFO that was still able to provide functional benefits including reduced walking energy cost and increased SSWS [26, 28, 44, 47]. This mismatch between improvements in functional and biomechanical variables is likely due to the fact that post-stroke gait patterns are heterogeneous, and individuals post-stroke may reorganize these already heterogenous gait patterns in a vast variety of ways when using the PD-AFO. Furthermore, these reorganized gait patterns may consist of a large number of small biomechanical adaptations that are hard to detect. This diversity of biomechanical gait patterns highlights the importance of evaluating outcome variables on an individual basis when prescribing PD-AFOs and other assistive devices for individuals post-stroke.

### Orthosis satisfaction

Our hypothesis that orthosis satisfaction would improve while wearing the PD-AFO compared to their SOC AFO was supported, as evidenced in the QUEST 2.0 and OPUS scores. Survey scores from this study are difficult to compare to published studies that also used these surveys since they were modified in this study to eliminate irrelevant questions. The significant improvement in participants’ satisfaction with the PD-AFO over their SOC AFO is compelling evidence that stiffness-customized PD-AFOs are favorably received by individuals post-stroke. Furthermore, the distribution of satisfaction with the PD-AFO compared to the SOC AFO is notable (Fig. 3). In particular, the QUEST 2.0 showed all participants were highly satisfied with the PD-AFO, but SOC AFO satisfaction varied drastically. Similar, although slightly less distinct, results were also seen with the OPUS. While a “new device” bias may have influenced these results to some extent (i.e. participants may believe the new device is supposed to be better, so they like it more), this finding is still notable. Overall, this positive response to the PD-AFOs is relevant for clinical work as it may indicate a greater willingness for individuals post-stroke to utilize similar PD-AFOs compared to traditionally prescribed devices.

### Limitations

While this study provides important insights to the benefits of stiffness-customized PD-AFOs for individuals post-stroke, some limitations exist. One potential limitation is the use of mechanical COT instead of the more commonly measured metabolic cost of walking. However, increased metabolic cost and increased mechanical work have been found to be related to one another in post-stroke gait [9, 10, 12, 48], thus changes in mechanical COT may indicate changes in metabolic cost. Secondly, all COT and biomechanical variable measurements were taken at the same baseline speed that participants walked at during their evaluation visit. COT and many biomechanical variables are speed dependent [35], so a consistent speed was required to compare the variables of interest across conditions. However, this method may have obscured speed-related benefits conferred by the PD-AFO. Future work should explore potential speed-related effects of PD-AFO use. Thirdly, it has been established that adapting gait patterns to treadmill walking takes approximately six to ten minutes [49]. However, this was not possible due to participants’ limited physical capacity to walk on the treadmill for such an extended period without noticeable fatigue. Additionally, this study only investigated one stiffness level for each participant. While this stiffness level proved to be beneficial for many, a different PD-AFO stiffness may have enabled even greater benefits. Future research should examine the effect of different PD-AFO stiffness levels on post-stroke gait function and mobility. Shoe type and design were also not controlled during this study. While many participants wore some form of athletic shoes, the differences in their designs may have influenced the results. Lastly, this study required participants to be able to walk without an orthosis or only with an orthosis that provided no plantar flexor assistance as this baseline data was needed to drive customization of the PD-AFO stiffness. While a diverse sample of participants was still captured in this study, this inclusion criteria certainly limited the study population to some extent, and therefore the results cannot be generalized to individuals who do not meet said criteria. Future work should assess alternative methods for customizing PD-AFO stiffness that does not require a No AFO baseline gait analysis.

## Conclusion

This study is the first, to our knowledge, to show that PD-AFOs with stiffness customized to make up for individual’s level of plantar flexor weakness have the capacity to reduce mechanical COT and improve walking speed for individuals post-stroke. Since these outcomes are key goals of post-stroke rehabilitation (50), this study demonstrated that stiffness-customized PD-AFOs have the potential to improve functional mobility for many individuals post-stroke. Notably, however, individual-level analysis revealed there is variability among participants, with these positive outcomes present in approximately half of the study population. Additionally, key biomechanical variables did not consistently improve with PD-AFO use. With the PD-AFOs showing positive improvements in COT and SSWS for a subset of participants, this work provides evidence of the efficacy of stiffness-customized PD-AFOs for many individuals post-stroke and lays the foundation for future work that can ultimately enable the effective prescription of PD-AFOs for patients post-stroke in clinical practice. Next steps should aim to identify participant characteristics that indicate for whom the stiffness-customized PD-AFO is effective. Additionally, future studies should work to identify the underlying mechanisms causing the COT reduction and whether individuals post-stroke improve their COT and SSWS with the PD-AFO after gaining greater familiarity with the brace through acclimation or training.

### Supplementary Information

Supplementary Material 1.

## Data Availability

Requests for data access can be sent to the corresponding author.

## Abbreviations

AFO: Ankle–foot orthosis
COT: Cost of transport
CLES: Common language effect size
PD-AFO: Passive-dynamic ankle–foot orthosis
SOC AFO: Standard-of-care ankle–foot orthosis
SSWS: Self-selected walking speed
