# Supplementary material for: Customized passive-dynamic ankle–foot orthoses can improve walking economy and speed for many individuals post-stroke
Source: J Neuroeng Rehabil. 2024 Jul 29;21:126. doi: 10.1186/s12984-024-01425-7 (PMC11285468; doi:10.1186/s12984-024-01425-7)
Supplement: Supplementary file 1 — Supplementary Material 1. [file 12984_2024_1425_MOESM1_ESM.docx]

**Supplementary Table 1.** Count of potential participants rejected for each inclusion/exclusion criterium.

| Inclusion/Exclusion Criterium | Count |
| --- | --- |
| Bilateral paresis caused by one or more strokes | 18 |
| Never prescribed a brace | 13 |
| Neurologic conditions other than stroke | 8 |
| Overall health concerns | 5 |
| Inability to walk for 2 minutes without assistance from another person during daily living | 2 |
| Cannot walk without a rigid orthosis | 2 |
| Insufficient cardiovascular health | 2 |
| Limited ROM | 1 |
| Exact reason not specified | 1 |
| Ataxic gait | 0 |
| Inability to walk outside the home prior to stroke | 0 |
| Total joint replacement or other orthopedic problems in the lower limb or spine that limited walking ability | 0 |
| Total | 52 |
